# Virucidal Efficacy of Olanexidine Gluconate as a Hand Antiseptic Against Human Norovirus

**DOI:** 10.1007/s12560-020-09422-4

**Published:** 2020-03-02

**Authors:** Kaoru Imai, Akifumi Hagi, Yasuhide Inoue, Mohan Amarasiri, Daisuke Sano

**Affiliations:** 1 Naruto Research Institute, Research and Development Center, Otsuka Pharmaceutical Factory, Inc., Naruto, Tokushima 772-8601 Japan; 2 Department of Frontier Science for Advanced Environment, Graduate School of Environmental Studies, Tohoku University, Aoba 6-6-06, Aramaki, Aoba-ku, Sendai, Miyagi 980-8579 Japan; 3 Department of Civil and Environmental Engineering, Graduate School of Engineering, Tohoku University, Aoba 6-6-06, Aramaki, Aoba-ku, Sendai, Miyagi 980-8579 Japan

**Keywords:** Human norovirus, Inactivation, Olanexidine, Antiseptic, RT-qPCR

## Abstract

Human noroviruses are the major cause of non-bacterial acute gastroenteritis worldwide. Since no therapeutic agent has been proven to prevent human norovirus infection yet, preventive healthcare interventions to block the infection routes play an important role in infection control. One of the possible infection routes of human noroviruses are through contaminated hands, but no hand antiseptics have been proven effective. Olanexidine gluconate is a new biguanide compound that has already been approved for sale as an antiseptic for the surgical field in Japan. A new hand antiseptic was developed using olanexidine gluconate in this study, and its virucidal efficacy against human noroviruses was evaluated using modified RT-qPCR that can account for genome derived from intact viruses using RNase A and photo-reactive intercalators. We tested the virucidal efficacy of five materials; two olanexidine gluconate antiseptics (hand rub formulation and surgical field formulation), two kinds of ethanol solutions at different pH (approx. 3 or 7), and a base component of olanexidine gluconate hand rub formulation against 11 human norovirus genotypes by culture-independent methods. The infectivity of murine norovirus (MNV), a surrogate for human norovirus, was significantly reduced after use of the antiseptics. The olanexidine gluconate hand rub demonstrated the strongest virucidal efficacy against human norovirus among the five tested materials. This study showed that olanexidine gluconate has the potential to become a strong tool for the prevention of human norovirus infection.

**Electronic supplementary material:**

The online version of this article (10.1007/s12560-020-09422-4) contains supplementary material, which is available to authorized users.

## Introduction

Noroviruses are a non-enveloped and single-stranded positive-sense RNA virus belonging to the family *Caliciviridae*, and its particle is an icosahedron with a diameter of 38 nm (Adams et al. 2016). Of the ten genogroups of norovirus, genogroups I (GI), GII, GIV, GVIII, and GIX can infect humans, and GIII, GV, GVII, and GX can infect bovine, mice, dogs, and bats, respectively, (Chhabra et al. 2019). Infectivity of human norovirus is considered to be very strong; fewer than ten viral particles have been reported adequate to establish infection (Feng et al. 2011). Human noroviruses cause acute gastroenteritis in medical facilities, schools and restaurants, and the economic burden of the illness is $4.2 billion and $60.3 billion in direct medical and indirect societal costs per year, respectively (Bartsch et al. 2016). Varied infection vehicles of human norovirus have been reported, including shellfish (Campos and Lees 2014), fruits (Berger et al. 2010) and other foods such as dried laver seaweed (Kusumi et al. 2017). Human noroviruses grow in small intestinal epithelial cells after a latency period of 12 to 48 h and cause symptoms such as abdominal pain, diarrhea, nausea and vomiting (Dolin et al. 1972). Usually the symptoms disappear within 2 days, but they may be severe depending on the physical strength and hygienic condition of patients (Fischer Walker et al. 2012; Liu et al. 2012). Human norovirus particles released from the feces of infected people are resistant to drying and heat, and maintain infectivity in natural environments, which poses a risk of secondary infection (Barclay et al. 2015; Lopman et al. 2012; Robilotti et al. 2015). Since no therapeutic agent or vaccine has been proven effective against human noroviruses, hygienic interventions, including hand washing, are regarded important for control of the infection (Barclay et al. 2015; Park et al. 2014; Park and Sobsey 2011) (Table 1).

**Table 1:** Genotype and viral titer of norovirus positive stool samples in this study

Genogroup	Genotype	Quantity^a^ (Log_10_ RNA copies/well)
Human norovirus GI	GI.2	4.45
	GI.3	5.75
	GI.4	4.85
	GI.6	4.07
	GI.7	5.75
Human norovirus GII	GII.2	4.52
	GII.4 Den Haag 2006b	4.82
	GII.10	4.17
	GII.12	4.56
	GII.14	4.29
Human norovirus GIV	GIV.1	6.16

^a^This value was RNA copies number of 100-fold diluted solution of test virus solution. This measurement was carried out prior to the virucidal activity test in order to confirm the concentration of the stool samples

Olanexidine gluconate is a novel antimicrobial compound developed by Otsuka Pharmaceutical Factory, Inc. and has been used in Japan clinically for preoperative skin preparation [Product name: Olanedine Antiseptic Solution 1.5% (OLG)] (Hagi et al. 2015; Inoue et al. 2015; Nakata et al. 2017). The virucidal efficacy of OLG against feline calicivirus at 60-s and 10 min were 0.60, and 2.85, respectively (unpublished data). Because European Committee for Standardization states that “the product shall demonstrate at least a decimal log reduction of 4 in virus titre” (EN 14476 2013), the virucidal activity of OLG against a non-enveloped virus is not enough as well as other biguanide compounds (Iwasawa et al. 2012; Sickbert-Bennett et al. 2005). The mode of action of OLG is similar to that of chlorhexidine gluconate, a biguanide antiseptic, but OLG has also been confirmed to have a stronger protein denaturation effect than chlorhexidine gluconate (Hagi et al. 2015).

The objective of this study is to develop a new olanexidine-containing formulation for hand hygiene (olanexidine gluconate hand rub; OLG-HR) in anticipation of the protein denaturation activity of OLG for destroying the capsid protein of human noroviruses. In OLG-HR, which is an improved version of OLG, ethanol was added to provide quick drying, and the pH was adjusted to be alkaline to enhance the capsid denaturation effect. The virucidal efficacy of five antiseptics including OLG-HR against eleven different genotypes of human norovirus was evaluated by a culture-independent method (RT-qPCR) using RNase A and photo-reactive intercalator PMAxx (propidium monoazide) pretreatment step. The virucidal effects of OLG-HR were compared with those of ethanol for disinfection (EtOH), acidically adjusted ethanol (pH 3) for disinfection (EtOH-A), base ingredient excluding olanexidine gluconate from OLG-HR (Base), and OLG (Table 2). EtOH was used as a test antiseptic because hand sanitizers containing ethanol has been recommended for the disinfection of hands by the Centers for Disease Control and Prevention (CDC) and World Health Organization (WHO) (Boyce and Pittet 2002; WHO 2009). EtOH-A was used because of its high inactivation efficiency against surrogate viruses, although its efficacy for human noroviruses were unknown (Park et al. 2010). The disinfection efficiency of OLG-HR and other four antiseptics against multiple genotypes within GI, GII and GIV of noroviruses were compared.

**Table 2:** Details of antiseptics evaluated in this study

Test material	Active ingredient		pH
	Name	Concentration of olanexidine gluconate (%)	
OLG-HR	Olanexidine gluconate/ethanol (70%)	1.5	9.5
EtOH	Ethanol (76.9–81.4%)	–	Approx. 7
EtOH-A	Ethanol (76.9–81.4%)	–	Approx. 3
Base	Ethanol (70%)	–	9.5
OLG	Olanexidine gluconate	1.5	5

## Results

### Virucidal Efficacy of Test Materials Against Murine Norovirus by Modified RT-qPCR and Plaque Assay

Since we have no simple in vitro culture method for human noroviruses, it is difficult to evaluate the correlation between the value of RT-qPCR and the virus infectivity. Therefore, we compared the results from the modified RT-qPCR and the plaque assay using murine norovirus (MNV) (Table 3). The virucidal efficiency of each test material was similar except for OLG. The log_10_ reduction values (LRVs, ± SD) of OLG at 30- and 60-s contact time evaluated using the plaque assay were 2.35 (± 0.22) and 2.73 (± 0.80), and those using the modified RT-qPCR assay were 1.90 (± 0.14) and 1.83 (± 0.42), respectively. Log reduction obtainable by the other four test materials containing ethanol were between 3.90 and 4.04 at both contact times when modified RT-qPCR assay was used, and those evaluated by the plaque assay were > 4.58.

**Table 3:** Virucidal efficacy of test materials against murine norovirus S99 by modified RT-qPCR and plaque assay

Test material	Reaction time (s)	Log reduction of modified RT-qPCR [log_10_ copies/well, mean ± SD, *n* = 3]	Log reduction by plaque assay [log_10_ PFU/mL, mean ± SD, *n* = 3]
OLG-HR	30	3.92 ± 0.21	> 4.58
	60	4.04 ± 0.18	> 4.58
EtOH	30	4.00 ± 0.25	> 4.58
	60	3.96 ± 0.17	> 4.58
EtOH-A	30	3.90 ± 0.16	> 4.58
	60	4.05 ± 0.22	> 4.58
Base	30	4.05 ± 0.21	> 4.58
	60	3.96 ± 0.20	> 4.58
OLG	30	1.90 ± 0.14	2.35 ± 0.22
	60	1.83 ± 0.42	2.73 ± 0.80

### Virucidal Efficacy of Test Materials Against Norovirus GI Genotypes

**Fig. 1:**
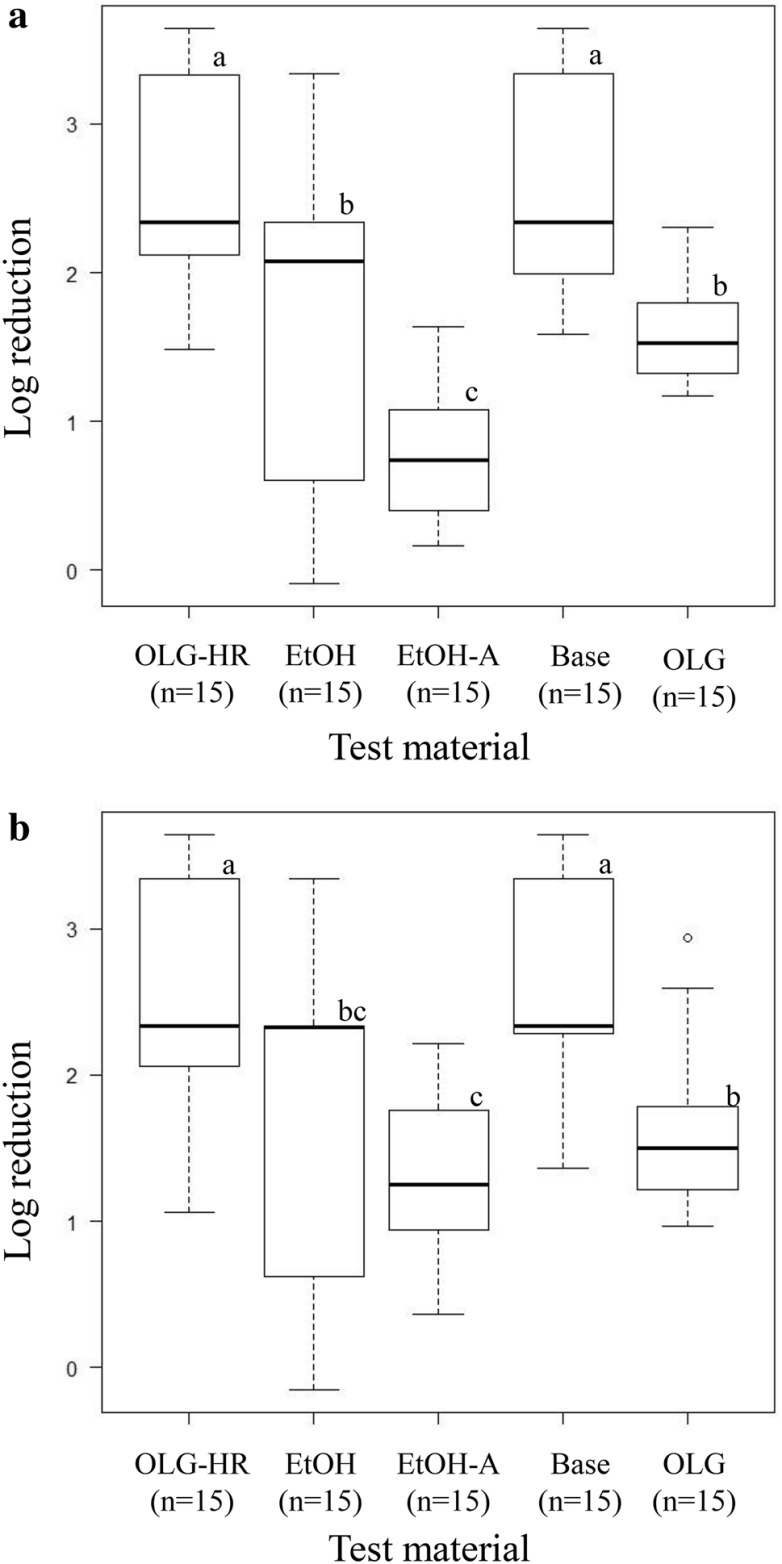
Comparison of virucidal efficacy of test materials against norovirus GI genotypes. Log reductions of GI.2, GI.3, GI.4, GI.6, and GI.7 were expressed collectively as box plots for 30- (**a**) and 60-s (**b**). The boxes not sharing a common letter differ significantly at *p* < 0.05 by Steel–Dwass test

The virucidal efficacy of test materials against norovirus GI genotypes was evaluated using the modified RT-qPCR assay. The RNA copy numbers (log_10_ RNA copies/well) of norovirus GI.2, GI.3, GI.4, GI.6, and GI.7 in PBS (negative control) were between 3.33 to 4.64, and the protein concentrations of five GI stool samples were between 2.19 and 14.61 mg/mL (Table S1). When OLG-HR was used, the mean LRVs of norovirus GI.4, GI.6, and GI.7 were higher than the quantification limit at the 30-s contact time (Table 4). The mean LRVs of norovirus GI.2 were 1.76 (± 0.18) and 1.70 (± 0.20) when the contact times with OLG-HR were 30- and 60-s, respectively, which were similar to the results of Base. The mean LRVs of EtOH against norovirus GI.4, GI.6, and GI.7 were > 2.34 log_10_ (higher than the quantification limit), > 2.33 log_10_, and 3.20 (± 0.36) log_10_ at 30-s, whereas those for norovirus GI.2 and GI.3 were 0.92 (± 0.31) and 0.10 (± 0.03) log_10_ even after 60-s. EtOH-A was not effective against norovirus GI.3, and the mean LRVs against the 4 other GI genotypes were between 1.11 and 1.81 log_10_ at 60-s (Table 4). The median LRVs of norovirus GI genotypes at 30-s contact time using OLG-HR, EtOH, EtOH-A, Base, and OLG were 2.34, 2.08, 0.74, 2.34, and 1.52 (Fig. 1a), and the 60-s contact time medians were 2.34, 2.33, 1.25, 2.34, and 1.50, respectively (Fig. 1b). The median LRV of norovirus GI genotypes was significantly higher when OLG-HR and Base were used at both contact times. The difference between OLG-HR and Base was not significant at both contact times (Fig. 1). There was no correlation between protein concentration of norovirus stool samples and LRVs for each test material both 30- and 60-s (Table S1 and Fig. S1).

**Table 4:** Virucidal efficacy of test materials against norovirus GI genotypes

Test material	Reaction time (s)	Log reduction [mean ± SD, *n* = 3]				
		GI.2	GI.3	GI.4	GI.6	GI.7
OLG-HR	30	1.76 ± 0.18	3.28 ± 0.54	> 2.34	> 2.33	> 3.34
	60	1.70 ± 0.20	> 3.64	> 2.34	> 2.33	> 3.34
EtOH	30	0.72 ± 0.16	0.01 ± 0.25	> 2.34	> 2.33	3.20 ± 0.36
	60	0.92 ± 0.31	0.10 ± 0.03	> 2.34	> 2.33	> 3.34
EtOH-A	30	0.99 ± 0.10	0.59 ± 0.07	0.38 ± 0.21	1.24 ± 0.17	0.80 ± 0.25
	60	1.16 ± 0.12	0.66 ± 0.02	1.11 ± 0.03	1.63 ± 0.16	1.81 ± 0.33
Base	30	1.75 ± 0.09	3.03 ± 0.49	> 2.34	2.03 ± 0.27	> 3.34
	60	1.80 ± 0.23	> 3.64	> 2.34	> 2.33	3.43 ± 0.01
OLG	30	1.51 ± 0.08	1.68 ± 0.28	1.26 ± 0.09	1.81 ± 0.40	1.57 ± 0.05
	60	1.63 ± 0.15	1.69 ± 0.36	1.18 ± 0.10	1.75 ± 0.30	1.66 ± 0.38

### Virucidal Efficacy of Test Materials Against Norovirus GII and GIV Genotypes

**Fig. 2:**
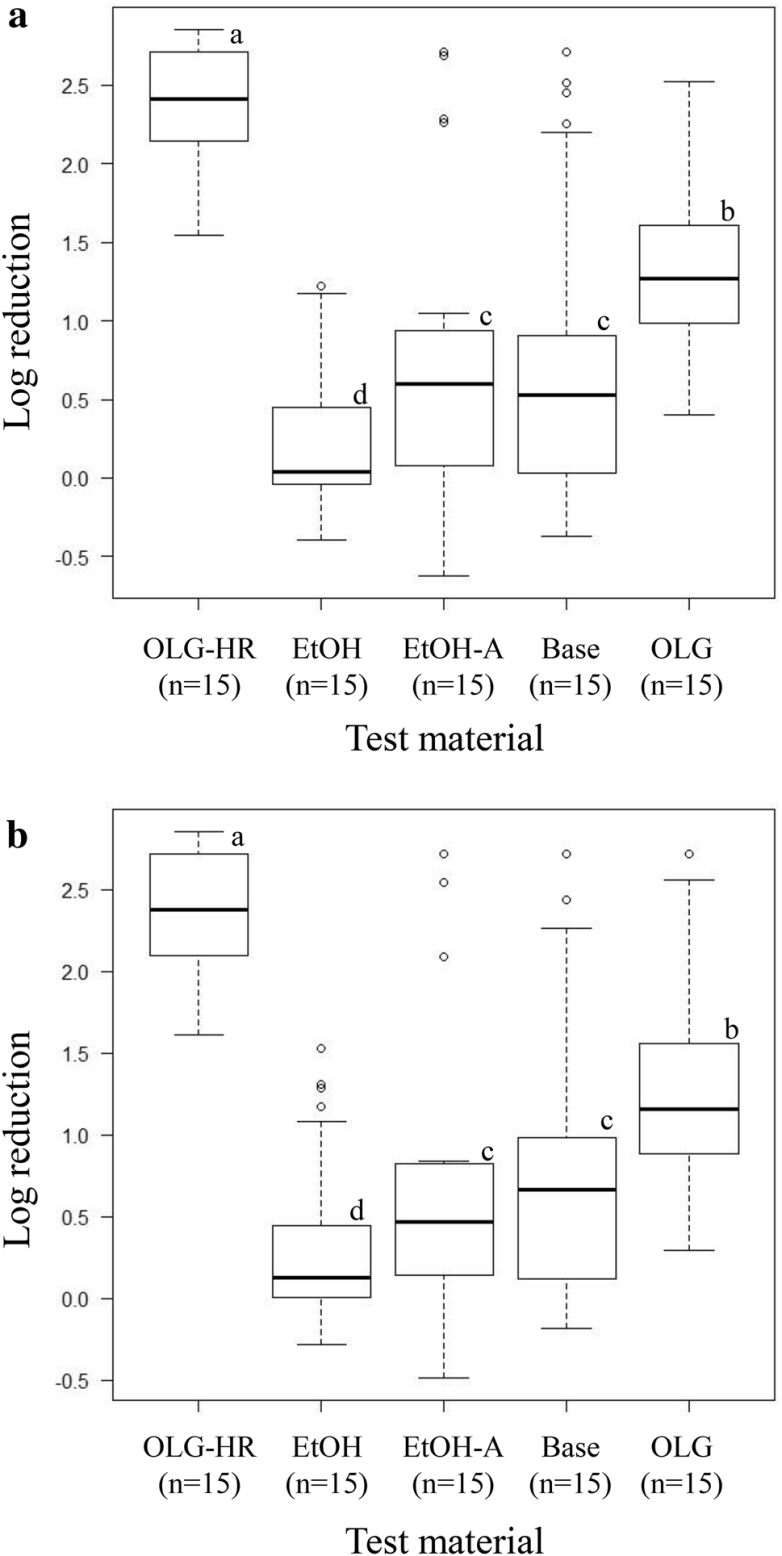
Comparison of virucidal efficacy of test materials against norovirus GII genotypes. Log reductions of GII.2, GII.4 Den Haag 2006b, GII.10, GII.12, and GII.14 were expressed collectively as box plots for 30- (**a**) and 60-s (**b**). The boxes not sharing a common letter differ significantly at *p* < 0.05 by Steel–Dwass test

The virucidal efficacy of hand sanitizers against norovirus GII and GIV genotypes was evaluated using the modified RT-qPCR assay (Table 5). The negative control (PBS) norovirus GII.2, GII.4 Den Haag 2006b, GII.10, GII.12 and GII.14 concentrations were between 3.14 and 3.85 log_10_, and the protein concentrations of five GII stool samples were between 2.65 and 18.78 mg/mL (Table S1). RNA copy numbers of norovirus GII.2, GII.4 Den Haag 2006b and GII.12 were below the quantification limit after 30-s contact with OLG-HR (Table 5). The LRVs of GII.10 and GII.14 at 30-s contact time were 1.78 (± 0.13) and 2.44 (± 0.11), respectively, which were the highest LRVs among all test materials. EtOH-A had a significant virucidal effect only on GII.4 Den Haag 2006b, but was ineffective for the other GII genotypes inactivation (Table 5). The median LRVs of norovirus GII genotypes were 2.41, 0.04, 0.60, 0.52, and 1.27 when exposed to OLG-HR, EtOH, EtOH-A, Base, and OLG for 30-s contact time, respectively (Fig. 2a). At 60-s contact time, these values were 2.37, 0.13, 0.46, 0.67, and 1.16, respectively (Fig. 2b). OLG-HR clearly had the strongest virucidal effect against tested norovirus GII genotypes, as in the case for norovirus GI genotypes. There was no difference in LRVs between the 30- and 60-s contact times (Fig. 2). There was no correlation between protein concentration of norovirus stool samples and LRVs with each test material after both 30- and 60-s (Table S1 and Fig. S1).

**Table 5:** Virucidal efficacy of test materials against norovirus GII and GIV.1 genotypes

Test material	Reaction time (s)	Log reduction [mean ± SD, *n* = 3]					
		GII.2	GII.4 2006b	GII.10	GII.12	GII.14	GIV.1
OLG-HR	30	> 2.85	> 2.71	1.78 ± 0.13	> 2.37	2.44 ± 0.11	> 4.24
	60	> 2.85	> 2.71	1.83 ± 0.14	> 2.37	2.63 ± 0.10	> 4.24
EtOH	30	0.16 ± 0.24	0.91 ± 0.12	0.02 ± 0.09	− 0.18 ± 0.19	0.15 ± 0.31	1.86 ± 0.18
	60	0.16 ± 0.03	1.13 ± 0.16	0.02 ± 0.15	− 0.04 ± 0.05	0.26 ± 0.23	1.92 ± 0.09
EtOH-A	30	− 0.21 ± 0.06	> 2.71	0.51 ± 0.20	0.27 ± 0.26	0.69 ± 0.34	> 4.24
	60	− 0.13 ± 0.03	> 2.71	0.53 ± 0.02	0.16 ± 0.17	0.76 ± 0.08	3.79 ± 0.08
Base	30	0.58 ± 0.09	2.10 ± 0.33	− 0.01 ± 0.17	− 0.05 ± 0.07	0.69 ± 0.16	2.28 ± 0.44
	60	0.76 ± 0.15	2.08 ± 0.27	0.06 ± 0.15	0.15 ± 0.05	0.70 ± 0.06	3.81 ± 0.20
OLG	30	0.89 ± 0.09	1.34 ± 0.25	1.42 ± 0.09	0.93 ± 0.29	2.29 ± 0.10	1.61 ± 0.37
	60	0.91 ± 0.14	1.12 ± 0.27	1.32 ± 0.13	0.88 ± 0.37	2.28 ± 0.19	1.90 ± 0.35

The negative control (PBS) virus and protein concentration of norovirus GIV.1 were 5.24 (± 0.18) log_10_ and 4.29 mg/mL, respectively (Table S1). Against the norovirus GIV.1 genotype, OLG-HR and EtOH-A had the strongest virucidal effect (> 4.24 log_10_). Also, the mean LRV of norovirus GIV.1 was higher at the 60-s contact time compared to 30-s when the Base was used. EtOH and OLG were less effective even at the 60-s contact time (Table 5).

## Discussion

In this study, we evaluated the virucidal effect of five antiseptics against eleven genotypes of human norovirus using a culture-independent method. Antiseptics OLG-HR, EtOH, EtOH-A, Base and OLG were used as test materials. There was no influence from the protein concentration of stool samples and residue of test materials for the norovirus log reduction. When OLG-HR was used, LRVs of norovirus GI.4, GI.6, GI.7, GII.2, GII.4 Den Haag 2006b, GII.12, and GIV.1 exceeded the quantification limit at 30-s, and the LRV of GI.3 exceeded the quantification limit at 60-s. The LRVs of norovirus GI.2, GII.10, GII.14 did not exceed the quantification limit, but the largest LRVs were obtained when OLG-HR was used. OLG-HR was the most effective against norovirus GII.2, which emerged in Japan in 2016–2017 season (Nagasawa et al. 2018). Meanwhile, Base and OLG were less effective compared to OLG-HR. Base had a strong virucidal effect against norovirus GI genotypes, as with OLG-HR, but was clearly inferior to OLG-HR against norovirus GII and GIV.1. The LRVs of norovirus GI genotypes were between 1.18 and 1.81 when OLG was used. EtOH was more effective against norovirus GI genotypes than EtOH-A, but was less effective against norovirus GII genotypes. Overall, it was evident that OLG-HR has the strongest virucidal effects among the five evaluated disinfectants.

Costantini et al. showed that a 70% ethanol solution can slightly disinfect norovirus GII.4 (Costantini et al. 2018). Another study reported < 0.5 log_10_ for human norovirus GII.2 and GII.4 by ethanol disinfection (Tung et al. 2013). Because of the complicated culture protocols of human noroviruses (Ettayebi et al. 2016), virucidal effects of disinfectants on human norovirus have been evaluated using surrogate viruses (Farkas et al. 2010; Hirneisen and Kniel 2013; Hoelzer et al. 2013). Unlike human norovirus, ethanol disinfection was effective for the above surrogate viruses (Cromeans et al. 2014; Kampf et al. 2005; Park et al. 2010). Our results indicate that unlike feline calicivirus, acidic ethanol is only effective against few human norovirus genotypes. Although the reason for this difference is not well understood, feline calicivirus, which causes upper respiratory disease, probably does not need to withstand environmental changes such as strong acidic conditions to establish infection (Gaskell et al. 2007). However, this study shows that results obtained from surrogate viruses do not necessarily mimic human norovirus, and, therefore, it is not clear how the sensitivity of these surrogate viruses to antiseptics can be compared with that of human norovirus.

Alternatively, culture-independent methods for investigating the infectivity of viruses have been used to evaluate the infectivity of enteric viruses (Sano et al. 2015). The culture-independent methods are based on RT-qPCR, and RNase or photo-reactive intercalators like PMAxx to distinguish undamaged (likely infectious) viral particles from damaged ones (Fraisse et al. 2018; Karim et al. 2015; Park et al. 2016; Randazzo et al. 2016). In considering the results of the culture-independent methods, the correlation with the results of culture-dependent methods is very important. In this study, prior to the evaluation of the virucidal efficacy of these antiseptics against human norovirus, we compared the LRVs obtained by a culture-dependent method (plaque assay) and a culture-independent method using culturable MNV (Gonzalez-Hernandez et al. 2012). Similar to previous studies, the susceptibility of MNV to ethanol-based antiseptics was confirmed using both methods (Belliot et al. 2008; Park et al. 2010). MNV was less susceptible to OLG irrespective of the virus quantification method than other test materials. These results indicate that the culture-independent method employed in this study may follow the reduction tendency of viral infectivity. Furthermore, the previously reported virucidal efficacy against norovirus GII.4 is similar to our test results of norovirus GII.4 Den Haag 2006b and therefore, our test results are considered to be reasonable (Costantini et al. 2018). On the other hand, the culture-independent methods tended to underestimate the MNV LRV compared to the culture-dependent method. Therefore, we considered that culture-independent methods can compare the relative virucidal efficacy of antiseptics or resistance of genotypes, but not show a true value of LRVs.

The results for virucidal effects on norovirus varied greatly among genogroups and genotypes. As for the tendency of difference in resistance by strain, norovirus GII seemed to be more resistant to antiseptics in general than norovirus GI. In addition, antiseptic resistances were not the same among genogroups; for example, the norovirus GI.2 genotype was relatively resistant among norovirus GII. Park et al. reported that the resistance of norovirus to ethanol disinfection differs between genotypes. However, the mechanism of antiseptic resistance has not yet been clarified (Park et al. 2016). Human norovirus tends to undergo higher genome mutation events than DNA-based microorganisms (Bull et al. 2005, 2010; 2012; Sanjuan et al. 2010). Furthermore, differences in several nucleotide sequences in open reading frames (ORF) 2 and ORF3 that encode the major capsid protein (VP1) and minor capsid protein (VP2) may influence disinfection resistance (Park et al. 2016; Rachmadi et al. 2018). Thus, the difference in the virucidal efficacy of OLG-HR and other antiseptics is thought to be due to differences in the capsid structure stability by a trivial difference in the amino acid sequence structure, which needs to be addressed in a future study.

The mechanism of action of virucidal efficacy of OLG-HR against human norovirus is not well understood, but it is known that at relatively high concentrations, olanexidine aggregates the cells through a protein-denaturing effect (Hagi et al. 2015; Sakagami et al. 2000). Furthermore, the virucidal effect of OLG-HR against non-enveloped viruses such as feline calicivirus and bacteriophage MS2 has been found to be stronger under basic conditions (data not shown). From these results, the mechanism of action of OLG-HR is expected to be the enhanced protein denaturation action of olanexidine under basic conditions by destroying the capsid structure and exposing the viral genome of human norovirus.

EtOH is the most fundamental component of hand sanitizers. EtOH was effective against norovirus GI.4, GI.6, and GI.7 of GI genotypes, but it was not effective against norovirus GII genotypes and GIV.1. On the other hand, EtOH-A was effective only against GII.4 Den Haag 2006b and GIV.1. Virucidal effects of acidic, neutral and basic ethanol (EtOH-A, EtOH and Base) were completely different confirming the influence of pH on the sensitivity of viruses to antiseptics. In addition, the pH dependent susceptibility varied among. It is known that the capsid structure of human norovirus is stable between pH 3 and 7, whereas at pH 10 the capsid structure loses its stability (Ausar et al. 2006; Cuellar et al. 2010). The pH of Base was adjusted to be 9.5, and it is possible to expect that the weak basic environment created by Base destabilizes the capsid structure and enhances the virucidal efficacy of olanexidine gluconate and ethanol. In fact, Base had greater virucidal activity against norovirus GI genotypes than EtOH and EtOH-A with efficacy equivalent to OLG-HR. The higher stability of human noroviruses to acidic ethanol may be related to their life cycle to establish infection. This is because human norovirus must withstand dramatic changes in gastrointestinal environmental conditions, such as pH 7 in the upper gastrointestinal tract and high acidity in the stomach until it reaches the small intestine at the site of infection.

In conclusion, OLG-HR has a stronger virucidal activity at 30- and 60-s contact time for human norovirus than EtOH, EtOH-A, Base, and OLG. OLG-HR has the potential to become the prevention and control tool against human norovirus infection.

## Material and Methods

### Test Viruses and Cells

MNV (strain S99) was purchased from Friedrich-Loeffler-Institut (Greifswald island Riems, Germany). RAW264.7 cells were used as the host of MNV (DS Pharma Biomedical, Osaka, Japan). RAW264.7 cells were grown with Dulbecco’s Modified Eagle’s Medium (DMEM; FUJIFILM Wako Pure Chemical Corporation, Osaka, Japan) containing 10% fetal bovine serum (Invitrogen, Carlsbad, CA, USA). MNV stocks were inoculated into host cells for 1 h and then cultured for 3 days after the medium change. Three freeze–thaw cycles were performed and centrifuged (3080×*g*, 4 °C, 30 min). After that, the supernatant was collected and ultracentrifuged at 111,000×*g* at 4 °C for 90 min using a Beckman 50.2 Ti rotor (Beckman Coulter, Inc., Fullerton, CA, USA). The pellet was suspended in PBS and passed through a filter with a pore size of 0.45 μm (Millipore, Bedford, MA, USA) and used as test virus solution for the virucidal tests. The viral genome quantities of the test human norovirus solution used in this study are shown in Table 1. Human noroviruses were obtained from stool specimens collected in Miyagi prefecture between 2006 and 2012. Each stool sample was diluted about 10 times with phosphate-buffer saline (PBS, FUJIFILM Wako Pure Chemical Corporation, Osaka, Japan) and centrifuged (4 °C, 10,000×*g*, 10 min), and the supernatant was recovered. The supernatant was passed through a filter with a pore size of 0.45 μm (Millipore, Bedford, MA, USA) and used as test virus solution for the virucidal tests.

### Suspension Test

The test materials were olanexidine gluconate hand rub (OLG-HR), Base component of olanexidine gluconate hand rub (Base), Onaledine Antiseptic Solution 1.5% (OLG, Otsuka Pharmaceutical Factory, Inc.), ethanol for disinfection (EtOH, Kenei Pharmaceutical Co., Ltd.) and acidic ethanol containing phosphoric acid (EtOH-A, Saraya Co., Ltd.), which is known to be effective for human norovirus surrogate virus (Table 2). Twenty micro-liters of test virus was mixed with 180 μL of test material (or PBS as a negative control) followed by incubation for 30- and 60-s at room temperature. After the incubation, 50 μL of the reaction solution was mixed with 450 μL of PBS. After that, the gel filtration column (MicroSpin S-400 h Columns; GE Healthcare, Piscataway, NJ, USA) treatment was performed immediately. The gel filtration column was used for the purpose of eliminating Olanexidine, which can influence the subsequent operation (Eterpi et al. 2010). Virucidal tests were performed in triplicate.

### Plaque Assay

Plaque assay was conducted as previously reported (Gonzalez-Hernandez et al. 2012). Host cells on 6-well plates were inoculated with 500 μL of tenfold dilution series of each reaction solution and incubated at room temperature for 1 h (*n* = 3). After removal of the reaction solution, an agar medium was added and incubated for 2 days at 37 °C. After that, 2 mL 0.01% neutral red solution (Sigma-Aldrich, St Louis, MO, USA) was added and incubated for 4 h at 37 °C. The neutral red solution was removed and the plaques were visually counted. Each assay was repeated three times. Log reductions of PFU were calculated, and results were expressed as mean ± SD.

### Modified RT-qPCR

Prior to RT-qPCR, the damaged or exposed viral genomes were removed using previously reported methods (Park et al. 2016; Randazzo et al. 2016). An aliquot (50 μL) of test- material-treated virus solutions was incubated with 56 μL of ultrapure water, 8 μL of RNase A (Promega, Madison, WI, USA), 2 μL of RNase buffer (Promega, Madison, WI, USA) for 1 h at 37 °C. The reaction was stopped by addition of 4 μL of RNase inhibitor (Invitrogen, Carlsbad, CA, USA), and then the mixture was incubated with 15 μL of PMAxx (Biotium, Hayward, CA, USA), 40 μL of PMA enhancer 5 × solution (Biotium, Hayward, CA, USA) and 21 μL of ultrapure water for 10 min in darkness and 15 min in visible light at room temperature.

After RNase and PMAxx treatment, MNV RNA of the reaction solutions was extracted using the QIAamp Viral RNA Mini Kit (Qiagen, Hiden, Germany). Reverse transcription was performed using High-Capacity cDNA Reverse Transcription Kit (Thermo Fisher Scientific, Rockford IL, USA). The RT reaction mixture was incubated at 25 °C for 10 min, 37 °C for 120 min and 85 °C at 5 min. Then, viral RNA was quantified by RT-qPCR as previously described (Kitajima et al. 2010) using an ABI PRISM 7500 Fast Real-time PCR System (Applied Biosystems, Foster City, CA, USA). RT-qPCR mix were performed in 25 μL reaction volume containing 12.5 μL TaqMan® Gene Expression Master Mix (Applied Biosystems, Foster City, CA, USA), 5 μL cDNA, 400 nM of each primer, and 300 nm of TaqMan probe. PCR conditions were initial denaturation at 95 °C for 10 min, 50 cycles of amplification with denaturation at 95 °C for 15 s, annealing and extension at 60 °C for 1 min. Technical replicates for each sample in each RT-qPCR were set to three (*n* = 3); the primers and probes used are listed in Table 6.

**Table 6:** RT-qPCR primers and probes used in this study

Target virus	Primer and probe	Seaquence (5′–3′)^d^	Polality	Location (bp)^e^	Length	References
Murine norovirus	MNV-S	CCGCAGGAACGCTCAGCAG	+	5028–5046	19	Kitajima et al. (2010)
	MNV-AS	CAGGCCGTCCCCATTCAGCC	−	5137–5156	20	
	MNV-TP^a^	ATGAGTGATGGCGCA	+	5062–5076	15	
Human norovirus GI	COG1F	CGYTGGATGCGNTTYCATGA	+	5291–5110	20	Kageyama et al. (2003)
	COG1R	CTTAGACGCCATCATCATTYAC	−	5354–5375	22	
	RING1(a)-TP^b^	AGATYGCGATCYCCTGTCCA	−	5021–5340	20	
	RING1(b)-TP^b^	AGATCGCGGTCTCCTGTCCA	−	5021–5340	20	
Human norovirus GII	COG2F	CARGARBCNATGTTYAGRTGGATGAG	+	5003–5028	26	Kageyama et al. (2003)
	COG2R	TCGACGCCATCTTCATTCACA	−	5080–5100	21	
	RING2-AL-TP^b^	TGGGAGGGSGATCGCRATCT	+	5048–5067	20	
Human norovirus GIV	Mon4F	TTTGAGTCYATGTACAAGTGGATGC	+	718–742	25	Trujillo et al. (2006)
	Mon4R	TCGACGCCATCTTCATTCACA	−	795–815	21	
	Ring4^c^	TGGGAGGGGGATCGCGATCT	+	763–782	20	

^a^5′- labeled with 6-carboxyfluorescein (FAM) and 3′-labeled minor groove binder (MGB)-non-fluorescent quencher (NFQ)

^b^5′-labeled with 6-carboxyfluorescein (FAM) and 3′-labeled with Carboxytetramethylrhodamine (TAMRA)

^c^5′-labeled with 6-carboxyfluorescein (FAM) and 3′-labeled with black hole quencher dye (BHQ)

^d^Mixed bases in degenerate primers and probes are as follows: Y, C or T; R, A or G; B, not A; N, any

^e^Nucleotide position based on MNV-1(GenBank accession no. AY228235), human norovirus GI (GenBank accession no. M87661), human norovirus GII (GenBank accession no. AF145896) and human norovirus GIV (GenBank accession no. AF414426)

For human norovirus, the RNA of the reaction solutions after RNase and PMAxx treatment was extracted using QIAcube (Qiagen, Hiden, Germany). Reverse transcription was performed using the iScript Advanced cDNA Synthesis Kit for RT-qPCR (Bio-Rad, Hercules, CA, USA). The RT reaction mixture was incubated at 42 °C for 30 min, and then at 85 °C for 5 min to inactivate the enzyme. RT-qPCR for human norovirus was performed using a CFX Connect Real-Time PCR detection system (Bio-Rad, Hercules, CA, USA). RT-qPCR mix were performed in 20 μL reaction volume containing 10 μL SsoAdvanced™ Universal Probes Supermix (Bio-Rad, Hercules, CA, USA), 5 μL cDNA, 400 nM of each primer, and 300 nm of TaqMan probe. PCR conditions were initial denaturation at 95 °C for 15 min to activate DNA polymerase, 40 cycles of amplification with denaturation at 95 °C for 15 s, annealing at 56 °C for 1 min and extension at 72 °C for 30 s (Kageyama et al. 2003; Trujillo et al. 2006). Technical replicates for each sample in each RT-qPCR were set to three (*n* = 3); the primers and probes used are listed in Table 6. The quantification limit of RT-qPCR of norovirus GI, GII, and GIV.1 were 10 copies/well. The PCR efficiency of norovirus GI, GII, and GIV.1 were 95.78%, 90.31%, and 95.02%, respectively.

### Statistics and Data Analysis for Virucidal Tests

Numerical values of each well of negative control (PBS) and test materials obtained by RT-qPCR were taken for a common logarithm. For the numerical values of the test materials of the three independent tests, log reduction was calculated compared to the negative control, and its average values and SD were calculated. When the numerical value of each well was below the limit of detection (10 copies/well in this test), the corresponding well was excluded from the subsequent calculation and expressed as BDL (below detection limit). If a BDL value was obtained twice or more in three technical triplicates, the numerical value in the test was BDL. If a BDL value was obtained twice or more in three independent tests, the RNA copies number of the test was BDL, and the LRV was set to be greater than the maximum detectable LRV (ex. > 3.5). In the statistical analysis, the numerical processing method was changed. That is, if the numerical value of each well was BDL, the value of the corresponding well was 10. The log reduction of each test material of the same genogroup was consolidated and expressed as a box plot. The upper and lower ends of the box and the horizontal line in the box indicate the first and third quantiles and the median value of data, respectively. The lower and higher ends of whiskers indicate the minimum and maximum value of the data, respectively. Isolated data points are outliers. Log reduction of each test material was analyzed with the Steel–Dwass non-parametric multiple comparison tests following the Kruskal–Wallis test. Data analysis was performed using Microsoft Excel 2010, and statistical analysis was performed using the statistical software SAS 9.2 (SAS Institute Japan) and EXSUS 7.7 software (CAC Excare Corporation).

### Correlation of Protein Concentration of Stool Samples and LRVs

This experiment was performed to confirm that fecal materials did not affect the efficacy of test materials in this study. The protein concentrations of stool samples were calculated by measuring the absorbance at 280 nm by NanoDrop (Thermo Fisher Scientific, Rockford IL, USA). Correlation between protein concentrations of stool samples and the mean of LRVs for each test material were evaluated with Spearman’s rank correlation coefficient (correlation coefficient (*r*_s_) and *p* values). The graphs were created using Microsoft Excel 2010, and statistical analysis was performed using the statistical software SAS 9.2 and EXSUS 7.7 software.

### Estimating the Effect of Antiseptic Residues on Modified RT-qPCR Reaction

This experiment was performed to confirm that antiseptic residues did not affect the efficacy of quantification test. Twenty micro-liters of PBS was mixed with 180 μL of test material (or PBS as a negative control) at room temperature. 50 μL of the reaction solution was mixed with 450 μL of PBS. After MicroSpin S-400 h treatment was performed, added 1/100 volume of stool samples of four genotypes and carried out the modified RT-qPCR. Technical replicates for each sample in each RT-qPCR were set to three (*n* = 3).

## Electronic supplementary material

Below is the link to the electronic supplementary material.

Supplementary file1 (DOCX 124 kb)
