# Supplementary material for: Virucidal Efficacy of Olanexidine Gluconate as a Hand Antiseptic Against Human Norovirus
Source: Food Environ Virol. 2020 Mar 2;12(2):180–90. doi: 10.1007/s12560-020-09422-4 (PMC7225205; doi:10.1007/s12560-020-09422-4)

Supplementary materials

Journal: Food and Environmental Virology

Title: Virucidal efficacy of olanexidine gluconate as a hand antiseptic against human norovirus

Authors: Kaoru Imai, Akifumi Hagi, Yasuhide Inoue, Mohan Amarasiri, Daisuke Sano

Corresponding author: Daisuke Sano, Ph.D., Department of Frontier Science for Advanced Environment, Graduate School of Environmental Studies, Tohoku University, Aoba 6-6-06, Aramaki, Aoba-ku, Sendai, Miyagi 980-8579, Japan

E mail: [daisuke.sano.e1@tohoku.ac.jp](mailto:daisuke.sano.e1@tohoku.ac.jp)

Table S1 Viral titer and protein concentration of norovirus positive stool samples in the virucidal tests

| Genogroup | Genotype | Quantity  (Log_10_ copies/well, mean ± SD) | Protein concentration  (mg/mL) |
| --- | --- | --- | --- |
| Human norovirus GI | GI.2 | 3.49 ± 0.05 | 2.44 |
|  | GI.3 | 4.64 ± 0.24 | 10.10 |
|  | GI.4 | 3.34 ± 0.17 | 5.34 |
|  | GI.6 | 3.33 ± 0.30 | 14.61 |
|  | GI.7 | 4.34 ± 0.51 | 2.19 |
| Human norovirus GII | GII.2 | 3.85 ± 0.29 | 2.65 |
|  | GII.4 Den Haag 2006b | 3.71 ± 0.29 | 3.01 |
|  | GII.10 | 3.14 ± 0.17 | 7.74 |
|  | GII.12 | 3.37 ± 0.19 | 6.34 |
|  | GII.14 | 3.85 ± 0.07 | 18.78 |
| Human norovirus GIV | GIV.1 | 5.24 ± 0.18 | 4.29 |

Table S2 Effect of antiseptic residues on modified RT-qPCR reaction

| Test material | Quantity  (Log_10_ RNA copies / well) | | | |  | Different from PBS | | | |
| --- | --- | --- | --- | --- | --- | --- | --- | --- | --- |
|  | GI.2 | GI.4 | GII.2 | GII.10 |  | GI.2 | GI.4 | GII.2 | GII.10 |
| PBS | 2.57 | 2.90 | 2.93 | 2.91 |  | - | - | - | - |
| OLG-HR | 2.71 | 2.86 | 2.96 | 2.91 |  | 0.15 | −0.04 | 0.03 | 0.00 |
| EtOH | 2.74 | 2.89 | 3.01 | 2.84 |  | 0.17 | −0.01 | 0.08 | −0.07 |
| EtOH-A | 2.66 | 2.89 | 2.99 | 2.90 |  | 0.09 | −0.01 | 0.05 | −0.01 |
| Base | 2.60 | 3.08 | 2.99 | 2.85 |  | 0.03 | 0.18 | 0.05 | −0.06 |
| OLG | 2.64 | 2.67 | 2.96 | 2.86 |  | 0.07 | −0.24 | 0.03 | −0.05 |

**Figure S1** The correlations between the protein concentrations of stool samples and log reductions. The correlations for 30- (a) and 60- (b) were analyzed by Spearman’s rank correlation coefficient


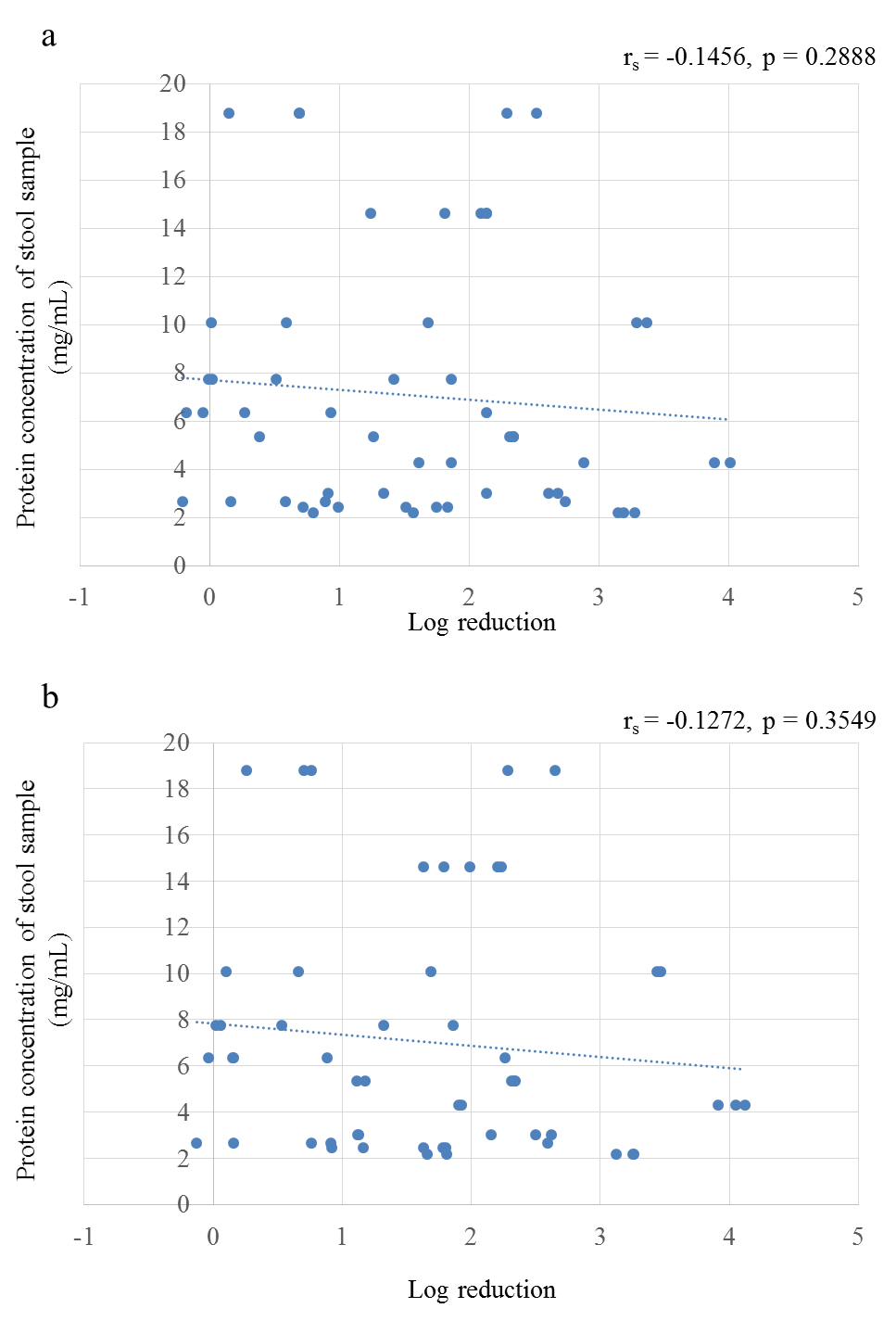

Supplement: Supplementary file 1 — Supplementary file1 (DOCX 124 kb) [file 12560_2020_9422_MOESM1_ESM.docx]
